# Experimental study on the buffering effects of urban trees group in dike-break floods

**DOI:** 10.1038/s41598-023-44024-7

**Published:** 2023-10-10

**Authors:** Shuyu Liu, Xiaolei Zhang, Zhiheng Xu, Jiankun Zhao, Boliang Dong

**Affiliations:** 1 School of Water Conservancy, North China University of Water Resources and Electric Power, Zhengzhou, 450046 China; 2 Guangdong Research Institute of Water Resources and Hydropower, Guangzhou, 510610 China; 3 State Key Laboratory of Water Resource and Hydropower Engineering Sciences, Wuhan University, Wuhan, 430072 China

**Keywords:** Fluid dynamics, Environmental sciences, Natural hazards

## Abstract

The process of dike-break flood propagation in typical urban street is highly complex. The presence of buildings and trees groups in urban street profoundly alters the flood dynamics, impacting the drainage capacity of the area. In this study, a generalized sink model representing a typical urban street was established, including trees groups, buildings, sidewalks, and stormwater drainage systems. The study measured the fluctuation of water levels within the street block and the pressure variation in the pressurized stormwater drainage network during the dike-break flood propagation. Furthermore, it conducted a comparative analysis to assess the influence of different arrangements of trees groups on the maximum water depth in buildings and the discharge capacity of the pressurized stormwater drainage network. Dike-break floods give rise to large-scale water leaps and the formation of thin layer water sheets near the buildings under the influence of buildings, water tank sidewalls, and tree groups. The water leap zones exhibit lateral migration and superposition on the sidewalks during the flood propagation, gradually dissipating and disappearing in the longitudinal direction of the street. In the presence of tree groups, the water levels significantly decrease in buildings and downstream street, while the discharge capacity of the pressurized stormwater drainage network shows a slight improvement as the road’s flood-carrying capacity increases. The pressure in the main pipes fluctuates due to the switching of the grate inlet drainage mode and the hydraulic transition process in the branch pipes. The research findings not only provide valuable validation data for numerical simulations but also offer theoretical guidance for urban flood management and landscape design.

## Introduction

In recent years, with the accelerated process of urbanization, the increasing prominence of issues such as the hardening of surface roads, destruction of natural water systems, and inadequate capacity of stormwater drainage networks becomes evident. The accelerated urbanization serves as the cause, while the hardening of surface roads, destruction of natural water systems, and inadequate capacity of stormwater drainage networks represent the resulting effects^1–4^.

During extreme heavy rainfall, water levels in nearby rivers rise rapidly, posing significant threats to the safety of people's lives and properties in the affected urban area. The stormwater drainage system in urban areas operates under pressurized flow conditions. If a breach occurs in the river, downstream street blocks become flood-prone areas during the flood propagation process. In general, breaches in river channels occur due to factors such as overflow, piping, and slope instability^5^. The discharge of the breach exhibits a trend of “initial increase, followed by decrease” during the widening process, reaching its maximum value when the breach rapidly expands downstream^6–8^. The dike-break wave propagates approximately in an elliptical shape within the flood-prone area, centered around the breach. Furthermore, the flood propagation time and peak velocity vary significantly across flood-prone areas with different roughness coefficients^9,10^. Specifically, when urban street serve as flood-prone areas during dike-break floods, the floodwater takes on complex flow patterns consisting of shock waves and reflected waves due to the intricate nature of residential areas. Within the urban area, water levels further rise as the flow area decreases, and near closely arranged buildings, the floodwater forms turbulent wake regions with a mixture of vortical structures^11–13^. Subsequently, some researchers have conducted in-depth studies on individual risk assessment, optimization of evacuation routes, and the impact of floods on underground spaces in urban flood events. They have analyzed the instability of vehicles in floods, the selection of evacuation paths for individuals, and improvement measures to enhance urban safety^14–19^.

Grate inlets, as a critical component of the stormwater drainage system, are designed to effectively control and manage the flow of rainwater. The discharge capacity of grate inlets is closely related to mitigating urban flood risks and safeguarding urban infrastructure and resident safety. Research on the discharge capacity of grate inlets is as follows: Under different inflow conditions, a single grate inlet exhibits two types of flow patterns: weir flow and submerged outflow. When the grate inlet experiences varying degrees of blockage, the discharge formula based on the Froude number can be adjusted by introducing a blockage coefficient^20,21^. The discharge of multiple grate inlets can be estimated using empirical formulas based on factors such as effective length ratio, effective width ratio, and open area ratio^22^. Additionally, the discharge capacity of grate inlets can be predicted through training using evolutionary genetic algorithms (GP models)^23^. Building upon this, Dong et al.^24^ developed a generalized sink model that incorporates typical urban street and stormwater drainage networks, along with their corresponding mathematical models. They further analyzed the grid size of grate inlets and the discharge formulas, and discovered that the stormwater drainage network can effectively reduce the surface water depth and flood wave velocity in the street blocks.

The stormwater drainage network is the most crucial infrastructure for mitigating urban waterlogging and reducing the impacts of flooding. It is also a key research focus in the development of sponge cities and resilient cities, highlighting its significant importance^25–29^. Numerical simulation of the stormwater drainage network has further advanced with the application of one-dimensional/two-dimensional coupled models, finite volume methods, unstructured mesh generation techniques, and hybrid parallel computing. These numerical simulation methods provide more accurate representations of surface water depth, hydraulic data such as pipe pressure and flow rates, and contribute to the refinement of stormwater management^30–33^. However, the stormwater drainage system is highly susceptible to blockages when improperly used and maintained. Fathy et al.^34^ conducted experimental studies to investigate the discharge capacity of trunk pipes under different levels and lengths of blockages. They found that the discharge capacity of the trunk pipes decreased by 15.11% under conditions where the blockage fill ratio along the pipe was 0.5. Tu et al.^35^ conducted long-term field investigations to analyze the extent of blockage caused by fallen leaves and other debris in the stormwater drainage system during summer and winter seasons. They further explored protective measures for the stormwater drainage system. In addition, Low Impact Development (LID) measures are considered to be more sustainable solutions for urban stormwater management due to their applicability in urban environments^36^. LID measures, such as rain gardens, permeable pavements, green roofs, and trees group, effectively store rainfall and provide longer retention times, resulting in delayed response and improved drainage efficiency of the stormwater drainage system^37–39^. Sañudo et al.^40^ explored the applicability of three mathematical models (the digital twin approach, the simplified spatial resolution approach, and the lumped approach) in relation to roof roughness coefficients and their suitability in the context of rainfall runoff processes.

In summary, during extreme heavy rainfall events, urban street are susceptible to dike-break floods, and the hydraulic conditions of the stormwater drainage system are complex and variable. The discharge capacity of the stormwater drainage system remains under overload conditions for an extended period. The influence of tree groups on the flood propagation process during dike-break floods requires further research and analysis. To address this, this study established a generalized sink model for typical urban street and investigated the flood propagation process in the presence of a pressurized stormwater drainage system. Qualitative analysis was conducted on the migration of water leap zones within the street, while quantitative analysis focused on the fluctuation of water levels and the discharge capacity of the pressurized stormwater drainage system.

## Experimental exploration

### Experimental apparatus

**Figure 1:**
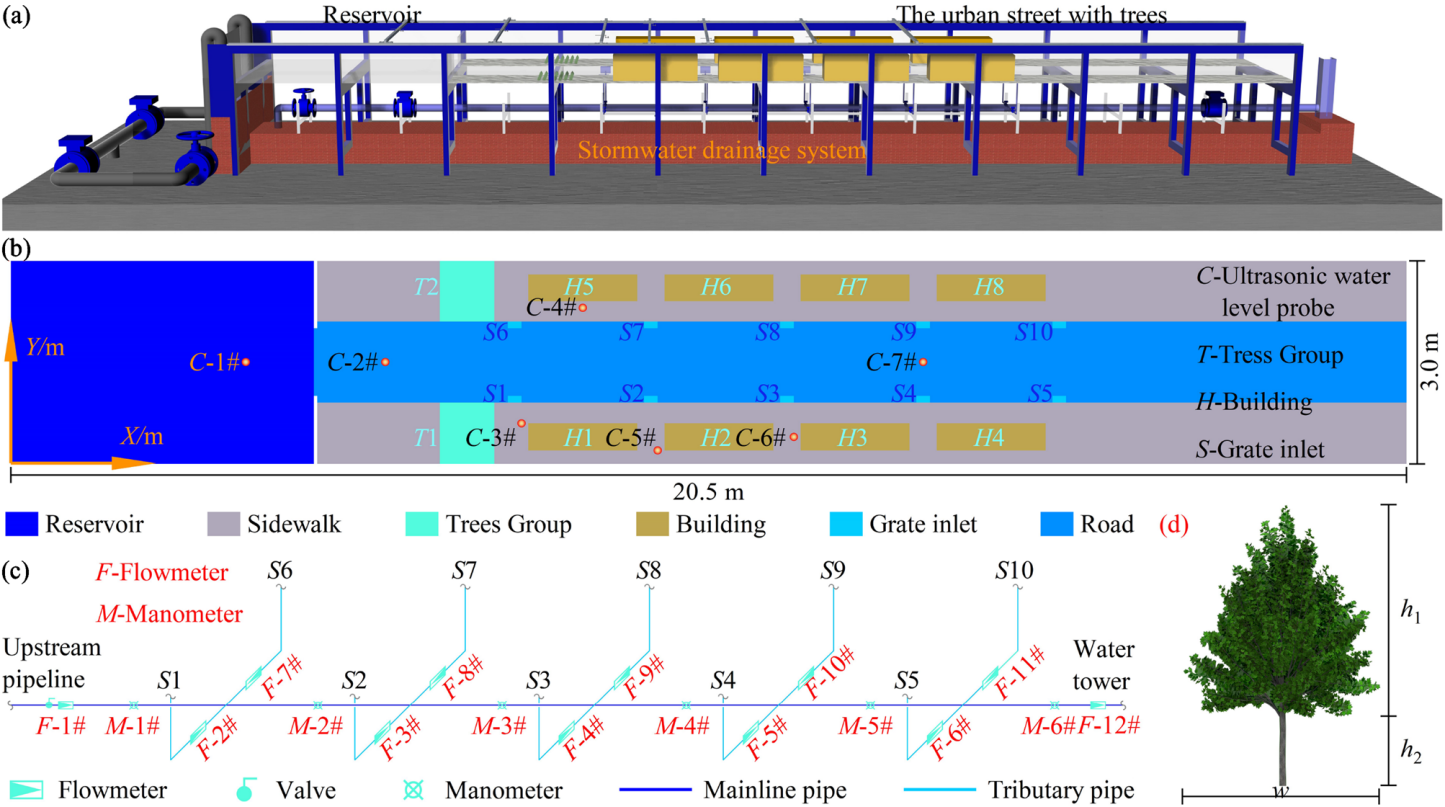
Schematic model and experimental apparatus setup: (**a**) Generalized sink model, (**b**) Plane layout inside the Tank, (**c**) Stormwater drainage system, and (**d**) Model tree.

The research employed a generalized sink model based on a typical urban street, which was divided into two layers, as shown in Fig. 1. The upper layer consisted of a large experimental glass water tank with a total length of 20.50 m, width of 3.00 m, height of 0.60 m, and a distance of 1.50 m from the ground. It included a reservoir chamber before the inlet, representing the pre-inlet reservoir, and the urban street. The lower layer comprised DN150 and DN25 glass pipes, representing the main pipes and branch pipes of the street block stormwater drainage system.

**Figure 2:**
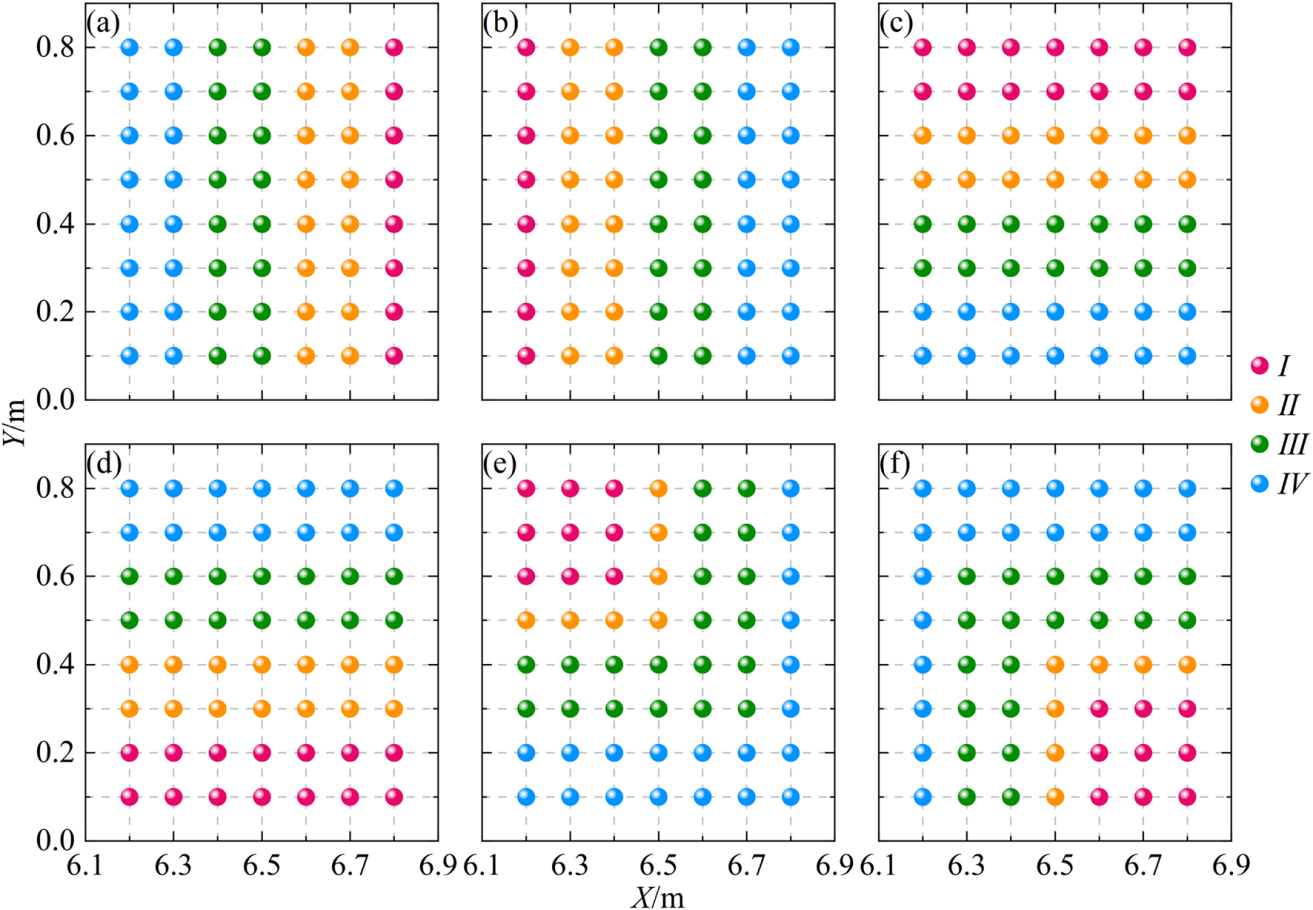
*T*1 Tree group arrangements: (**a**) EXP-2, (**b**) EXP-3, (**c**) EXP-4, (**d**) EXP-5, (**e**) EXP-6, and (**f**) EXP-7.

The reservoir has dimensions of 4.45 m in length and 3.00 m in width. It is supplied with water through two inlet pipes equipped with valves and flowmeters. In the middle of the reservoir's outlet, a rigid gate measuring 0.05 m in length and 1.00 m in width is installed. By rapidly lifting a baffle, dike-break floods can be created during the experiments. The urban street is designed according to typical Chinese street standards and consists of roads, grate inlets, sidewalks, buildings, and tree groups. The street is symmetrically distributed along the centerline *Y* = 1.50 m. Here are the specific dimensions and arrangements:

1. The road is 16.00 m long and 1.20 m wide.
2. There are 0.90 m wide sidewalks on both sides of the road, with a height difference of 0.01 m compared to the road.
3. Ten grate inlets, labeled as *S*1 to *S*10, are evenly spaced between the sidewalks and the road. Each grate inlet has dimensions of 0.20 m in length, 0.10 m in width, and a well depth of 0.14 m. The spacing between *S*1 and *S*2 is 1.80 m, and the coordinate of the bottom left corner of *S*1 is (7.30, 0.90).
4. Two rows of buildings, arranged in a 4 × 2 grid, are located on the sidewalks. Each building has dimensions of 1.60 m in length, 0.40 m in width, and 0.60 m in height. The spacing between *H*1 and *H*5 is 0.40 m, and the coordinate of the bottom left corner of *H*1 is (7.60, 0.20).
5. On the upstream side of the sidewalk, tree clusters with dimensions of 0.80 m in length and 0.90 m in width are placed. The tree species used in the model can be found in Table 1, and the arrangement of the tree clusters is shown in Fig. 2.

**Table 1:** Tree dimensions.

Type	*h*_1_/cm	*h*_2_/cm	*w*/cm
*I*	4.0	2.0	3.0
*II*	6.0	3.0	4.0
*III*	5.0	3.0	4.0
*IV*	7.5	3.5	5.5

The mainline pipe of the stormwater drainage system is located at a distance of 0.675 m above the bottom of the tank. Valves and flowmeters are used to control the water supply flow rate at the upstream end of the mainline pipe. A water tower is employed at the downstream end to modify the internal pressure of the pipe. The connection between the mainline pipe and the tributary pipe is achieved through a centered joint, and each tributary pipe corresponds to a grate inlet. Flowmeters are utilized to measure the changes in flow rate within the pipes.

### Experimental measuring apparatus

In order to investigate the buffering effect of tree groups on dike-break floods, ultrasonic water level probes *C*-2# to *C*-7# were used to monitor the water level changes during the flood propagation in the stree. Probe *C*-1# was utilized to measure the water level variations in the reservoir. The coordinates of each water level monitoring point are provided in Table 2. Additionally, flowmeters *F*-1# to *F*-12# and manometers *M*-1# to *M*-6# were employed to record the actual data of flow rates and pressures inside the pressurized stormwater drainage network during the dike-break flood propagation.Flowmeters *F*-1# and *F*-12# were installed at the inlet and outlet of the mainline pipe to monitor the overall inflow and outflow rates of the stormwater drainage system. Flowmeters *F*-2# to *F*-11# were positioned in the tributary pipes corresponding to grate inlets *S*1 to *S*10, respectively. Manometers *M*-1# to *M*-6# were evenly distributed along the bottom of the mainline pipe, with a vertical coordinate of 1.50 m. The horizontal coordinates of the manometers are 6.40, 8.40, 10.40, 12.40, 14.40, and 16.40 m.

**Table 2:** Measurement point location.

Measurement point	*X*/m	*Y*/m
*C*-1#	3.45	1.50
*C*-2#	5.50	1.50
*C*-3#	7.50	0.60
*C*-4#	8.40	2.30
*C*-5#	9.50	0.20
*C*-6#	11.50	0.40
*C*-7#	13.40	1.50

The ultrasonic water level probes are manufactured by Shangshui Company in China. They have a measurement frequency of 4 Hz and a measurement error of ± 0.2 mm. The flowmeters have a DC voltage output of 1–5 V and a measurement accuracy of 0.5 level. The manometers use high-precision diffusion silicon transmitters with a measurement range of − 0.1 to 0.6 MPa. They operate at temperatures ranging from − 20 to 85 °C, with a measurement frequency of 10 Hz and a response time of less than 1 ms. The measurement accuracy is at a level of 0.3.

### Experimental conditions and procedures

The study investigates the buffering effect of trees groups on dike-break floods by comparing different scenarios with and without trees groups. Based on Fig. 2, seven experimental conditions were established to compare the buffering effect of different trees groups on dike-break floods. Condition EXP-1 represents the control group without any trees group in the street, while conditions EXP-2 to EXP-7 correspond to the combinations labeled as a to f in Fig. 2. In each experimental condition, the inlet flow rate (*Q*_0_) of the stormwater drainage system is set at 10 L/s, and the reservoir water level (*H*) varies at 0.15 m, 0.25 m, and 0.35 m.

Prior to the start of the experiments, it is important to ensure that the mainline pipe is operating under pressurized flow conditions, while the tributary pipes maintain a certain water level. The rigid gates are closed using the stop boards, and the water level in the reservoir is adjusted to the desired storage height by regulating the valves and flowmeters. Once the water level in the reservoir stabilizes, the stop boards are instantaneously lifted to simulate the occurrence of a dike-break flood.

The sidewalks in this experiment are made of ceramic tiles, while the trees group is embedded in acrylic organic glass panels with the same dimensions. The roughness of the ceramic and glass surfaces is measured to be 0.03 mm and 0.02 mm, respectively. Dong et al.^41^ found in their model experiments that the propagation of the dike-break wave is hardly affected by such differences in roughness. Therefore, the experiment is expected to be minimally influenced by changes in materials. During the experiment, the occurrence of a dike-break flood is simulated by instantaneously lifting the stop boards. The feasibility of this step has been verified in previous model experiments conducted by Dong et al.^24^, taking into account the research work of Lauber et al.^42^ and von Hafen et al.^43^.

### Reproducibility of the experiment

Despite the feasibility of the crucial steps in the experiment being validated in previous research, it is necessary to measure the water level fluctuations caused by the dike-break flood, as well as the changes in flow rate and pressure within the stormwater drainage system. The measurement accuracy and response time of the experimental instruments need to be further analyzed. Additionally, the deflection and arrangement of the model trees also affect the measured data. Therefore, this study conducted a minimum of 3 repeated experiments for each condition to ensure the authenticity and repeatability of the experimental data, resulting in a total of 63 experiments. Taking the measured data of water level, pressure, and flow rate from multiple repeated experiments of EXP-4 with a reservoir water level of 0.35 m as an example, the Pearson correlation coefficient is calculated to analyze the correlation between the repeated experiments. The calculation formula is as follows:

1 $$R=\frac{\sum_{i=1}^{n}({X}_{i}-\overline{X })({Y}_{i}-\overline{Y })}{\sqrt{\sum_{i=1}^{n}{({X}_{i}-\overline{X })}^{2}}\sqrt{\sum_{i=1}^{n}{({Y}_{i}-\overline{Y })}^{2}}}$$

where *n* represents the number of data points in sample *X* or *Y*; $\overline{X }$ and $\overline{Y}$ represent the mean values of sample *X* and *Y, respectively*; and *X*_*i*_ and *Y*_*i*_ represent the measured data indexed by *i* in sample *X* and *Y*, respectively.

**Figure 3:**
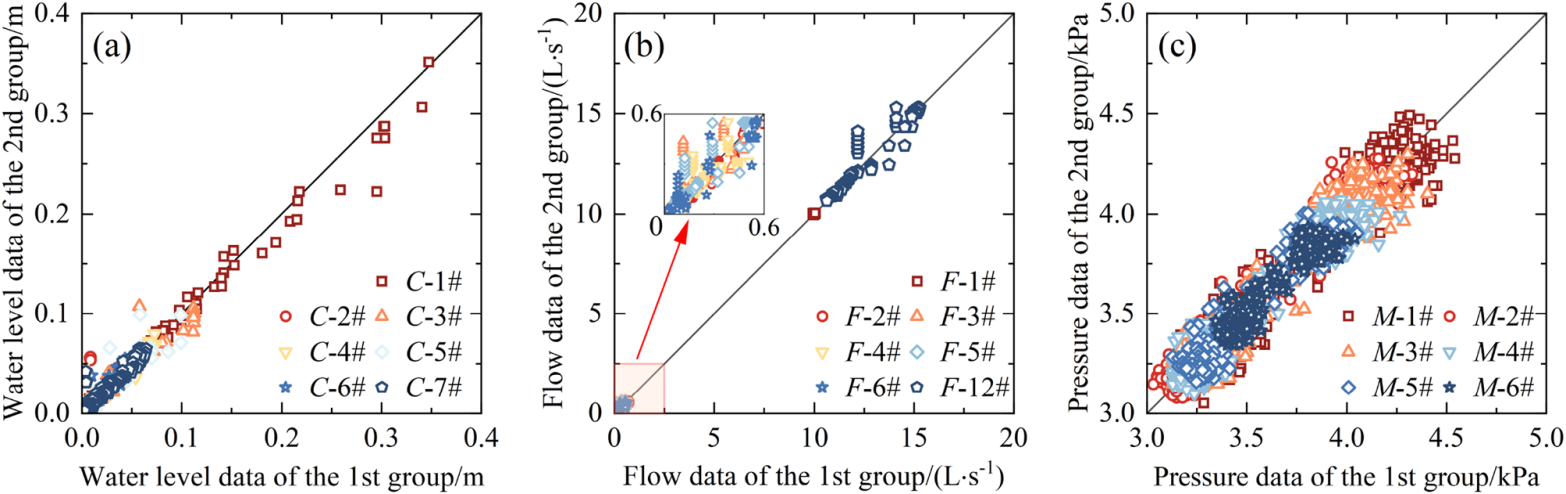
Comparison of Reproducibility Test Results: (**a**) Comparison of water level data, (**b**) Comparison of flow data, and (**c**) Comparison of pressure data.

Based on Fig. 3, it can be observed that the correlation coefficients (*R*) for the ultrasonic water level probes *C*-1# to *C*-7#, the manometers *M*-1# to *M*-6#, and the flowmeters *F*-1# to *F*-6# and *F*-12# range from 0.907 to 0.996. This indicates a high correlation in the measured data of both the water level fluctuations in the tank and the instantaneous variations in flow and pressure in the stormwater drainage system. The high correlation suggests accurate control of experimental conditions and repeatability of the experimental process, indicating the reliability and validity of the experimental results.

## Results and discussion

### Flood hydrodynamic characteristics

**Figure 4:**
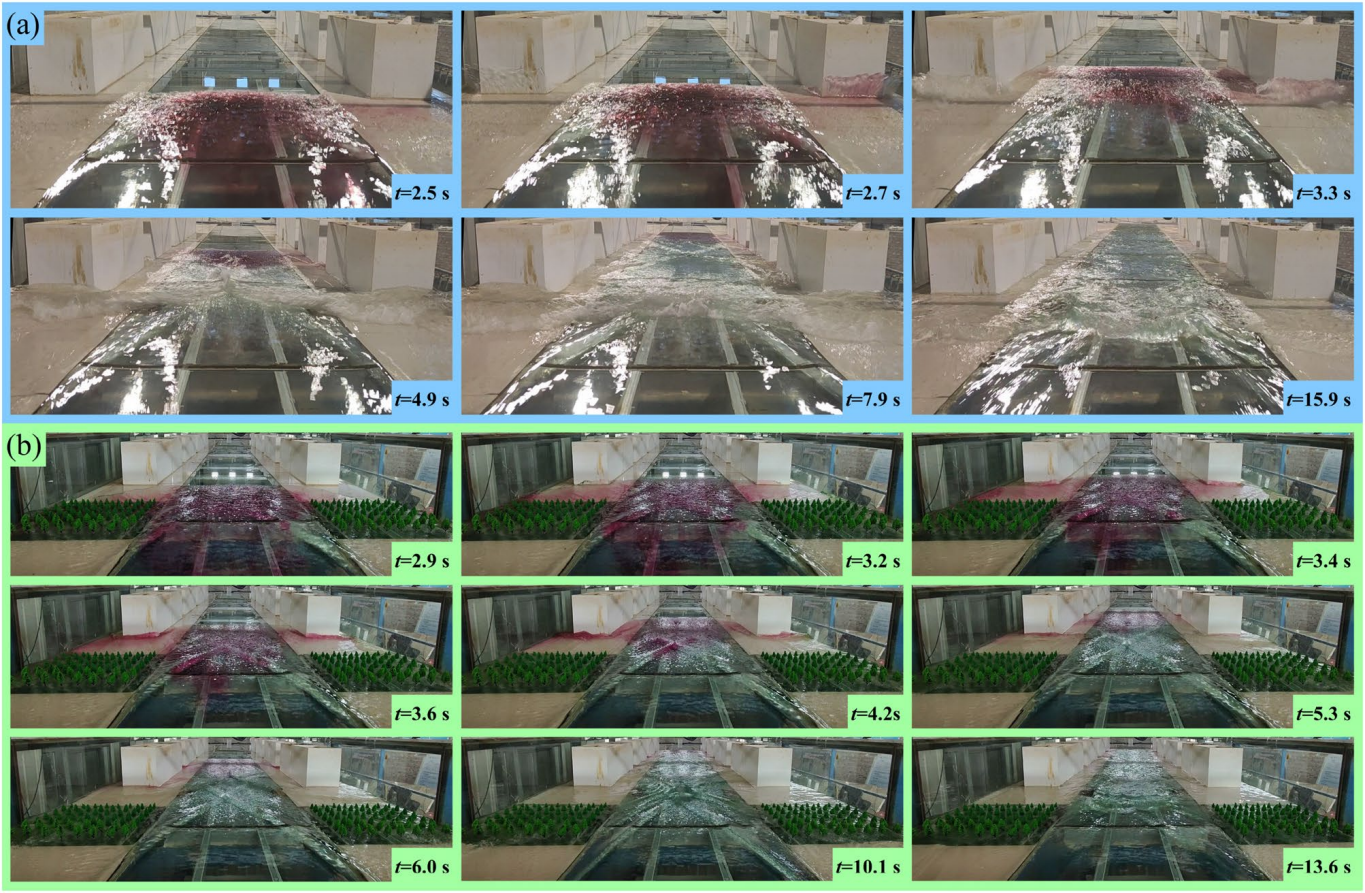
Change of flow regime in the urban: (**a**) The urban without tress, (**b**) The urban with tress.

**Figure 5:**
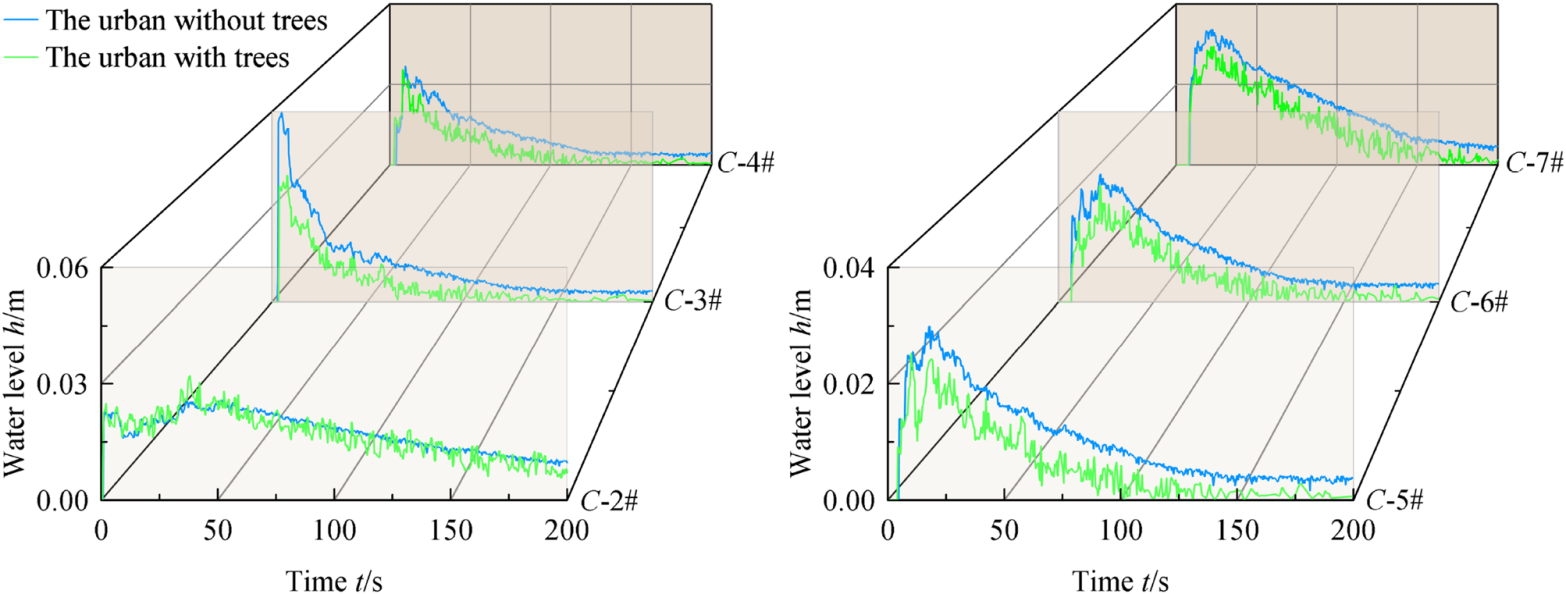
Fluctuations in Water Level at Measurement Points.

Taking the example of experimental conditions EXP-1 and EXP-2 with a reservoir water level height (*H*) of 0.15 m, the comparison of the progression of the floodwater in the presence and absence of tree groups in the street is shown in Fig. 4. The comparison of water level fluctuations at measurement points C-2# to C-7# in the urban is depicted in Fig. 5.

In the absence of tree groups in the urban block, when the gate of the breach is opened, the potential energy of the water in the reservoir is converted into the kinetic energy of the floodwater. The floodwater reaches the vicinity of buildings *H*1 and *H*5 within 2.5 s, and near these buildings, thin water sheets are formed initially between 2.7 and 3.3 s, which then roll and mix with a significant amount of air to form hydraulic jumps. The hydraulic jump region migrates from the sides of the water tank to the centerline of the road (*Y* = 1.50 m), and the merging of hydraulic jump regions on both sides of the water tank is completed at 4.9 s. After merging, the hydraulic jump region undergoes migration along the *X* direction. It initially migrates upstream to the furthest point of the urban block at 15.9 s, and then migrates downstream until it dissipates completely.

In the presence of trees in the urban street, the floodwaters climb the sidewalk and reach the vicinity of buildings *H*1 and *H*5 with a delay of 0.2 s compared to EXP-1. Additionally, the thin sheet of water formed near the buildings is significantly smaller compared to EXP-1. During the evolution of the floodwaters in the urban street, noticeable water leap areas form near the trees, along the walls of the water tank, and near the buildings. The water leap areas near the walls of the water tank and buildings complete their convergence and move upstream within the street at 10.1 s. They intersect again at 13.6 s, forming a water leap area that reaches the upstream end of the street. Finally, the water leap area migrates downstream until it dissipates completely.

Based on Fig. 5, further quantitative analysis can be conducted to examine the water level fluctuations during the flood progression in the urban street. Under different conditions, the water level fluctuations at each measurement point exhibit a similar overall trend.

Due to the migration of the water leap area within the urban street, the peak water level at point C-2# lags behind points C-3# to C-7#. Additionally, the presence of the tree group further reduces the flooded area within the water tank, resulting in an increase in the peak water level at point C-2# from 2.59 to 3.20 cm. After reaching peak water levels in a short period of time, the rate of water level decrease decreases with the increase in flood duration for C-3# ~ C-7#. The overall water levels in buildings and downstream neighborhoods decrease due to the energy dissipation effect of tree groups: ① After encountering the water-blocking effect of the tree groups, the peak water levels at the building measurement points show varying degrees of reduction, for example, the difference in peak water levels at C-3# and C-4# is 1.96 cm and 0.14 cm, respectively; ② The water level at downstream measurement point C-7# decreases by 0.5 cm during the flood propagation period.

### Discharge and pressure fluctuations in pressurized rainwater pipe network

**Figure 6:**
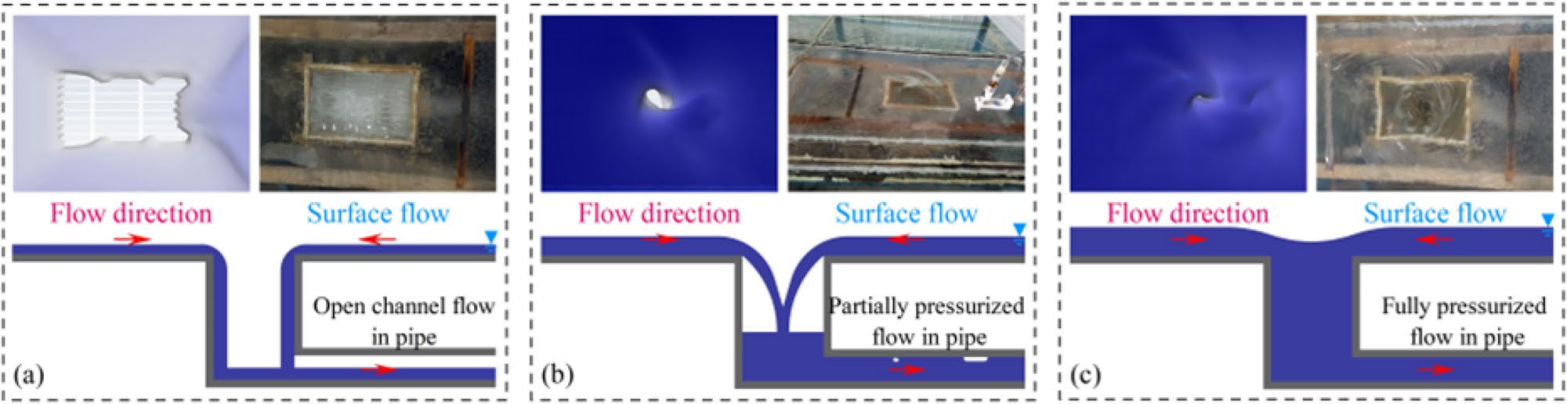
Discharge model of the grate inlet: (**a**) Weir flow pattern I, (**b**) Weir flow pattern II, and (**c**) Orifice flow.

The discharge of the grate inlet directly reflects the fluctuation in the overall flow rate of the pressurized stormwater drainage system. In previous research^20,44,45^, the discharge modes of the grate inlet have been classified into weir flow and orifice flow based on the water depth in front of the grate. Specifically, weir flow can further be categorized into two types depending on the state of the tributary pipe, either as non-pressurized flow or semi-pressurized flow, as illustrated in Fig. 6. Previous studies have mainly focused on analyzing the discharge capacity of the grate inlet under orifice flow conditions. It has been found that the discharge of the i-th tributary pipe in the urban area is related to the exponent ratio of the pressure difference between the upstream and downstream, with a ratio of 2:1, as shown in the following equation^46^:

2 $${Q}_{i}=aA\sqrt{g{h}_{i}}$$

where *Q*_*i*_ represents the discharge of the i-th tributary pipe in the pressurized stormwater drainage system in the urban area; *a* is the coefficient to be determined; *A* represents the effective cross-sectional area of the tributary pipe for flow; *g* is the acceleration due to gravity; and *h*_*i*_ represents the pressure difference between the upstream and downstream of the tributary pipe.

**Figure 7:**
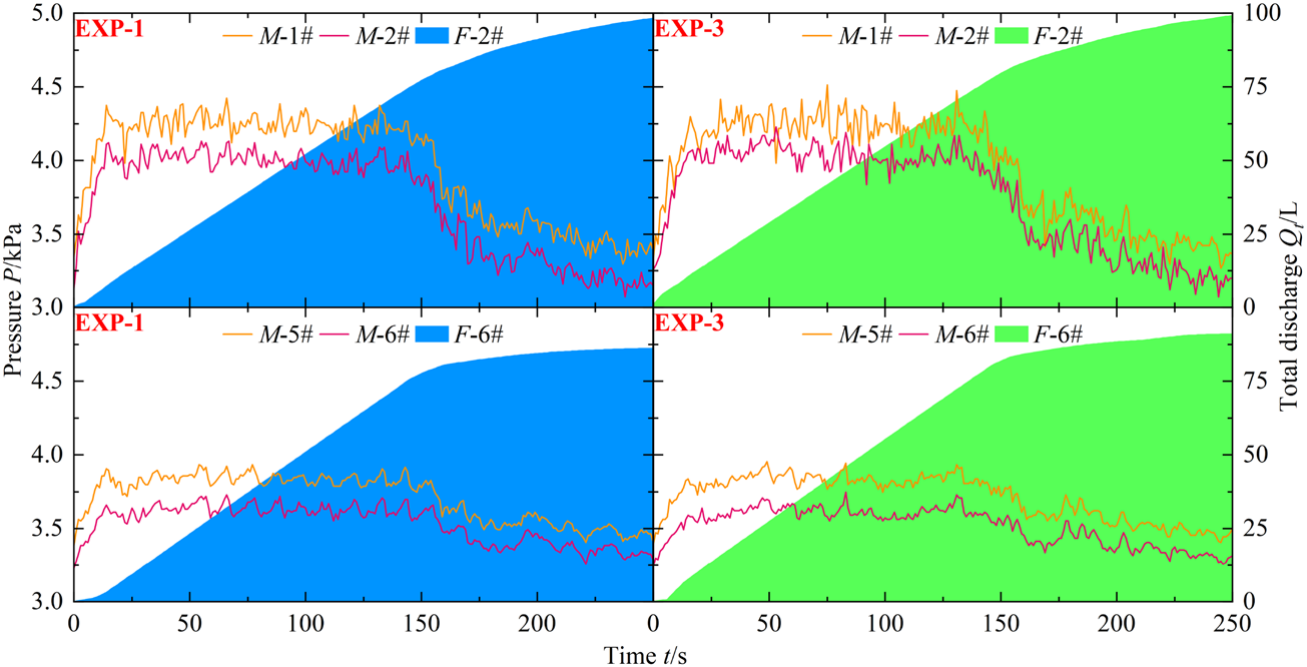
Fluctuations in discharge and pressure within the drainage network.

To further investigate the transition process of the discharge mode of the grate inlet during flood propagation, Fig. 7 illustrates the total discharge of the grate inlets *S*1 and *S*5, as well as the pressure fluctuations at the upstream and downstream of the mainline pipes connected to the tributary pipes *F*-2# and *F*-6#. The example is based on experimental conditions EXP-1 and EXP-3, with a reservoir water level of *H* = 0.25 m.

During the initial stage of flood propagation (0–30 s), the discharge mode of the grate inlets transitions rapidly from weir flow to orifice flow. The tributary pipes experience an instantaneous transition from no-pressure flow to full-pressure flow, resulting in an increase in flow velocity within the pipes. The pressure in the mainline pipes gradually increases after the convergence of the tributary flows. Despite the formation of large water leap areas during the flood propagation in the urban street, the influence of water level fluctuations on the discharge capacity of the grate inlets is negligible according to Eq. 2. As a result, the total discharge of the grate inlets linearly increases during the middle stage of flood propagation (30–150 s), while the pressure in the mainline pipes remains relatively constant. In the later stage of flood propagation (150–250 s), the discharge mode of the grate inlets transitions back from orifice flow to weir flow. The reduction in pressure difference between the upstream and downstream of the tributary pipes directly weakens the discharge capacity of the grate inlets, leading to a nonlinear increase in the total discharge. Meanwhile, the pressure in the mainline pipes gradually decreases.

Based on the previous discussion, it can be observed that the floodwater, hindered by the presence of tree groups, mainly propagates along the roads in the urban street. This results in a relatively larger total discharge of the rainwater network in EXP-3 compared to EXP-1. For example, the total discharge of rainwater at S5 inlet in EXP-1 and EXP-3 during the 0–250 s period is 86.10 L and 90.96 L, respectively. This further explains the decrease in water level downstream of the urban street when the discharge capacity of the rainwater network is enhanced. With the improved discharge capacity of the grate inlets due to the presence of tree groups, the pressure in the mainline pipes increases further. For instance, the peak pressure at pressure gauge M-1# in EXP-1 and EXP-3 is 4.42 kPa and 4.51 kPa, respectively.

### Impact of tree arrangements on urban protection

**Figure 8:**
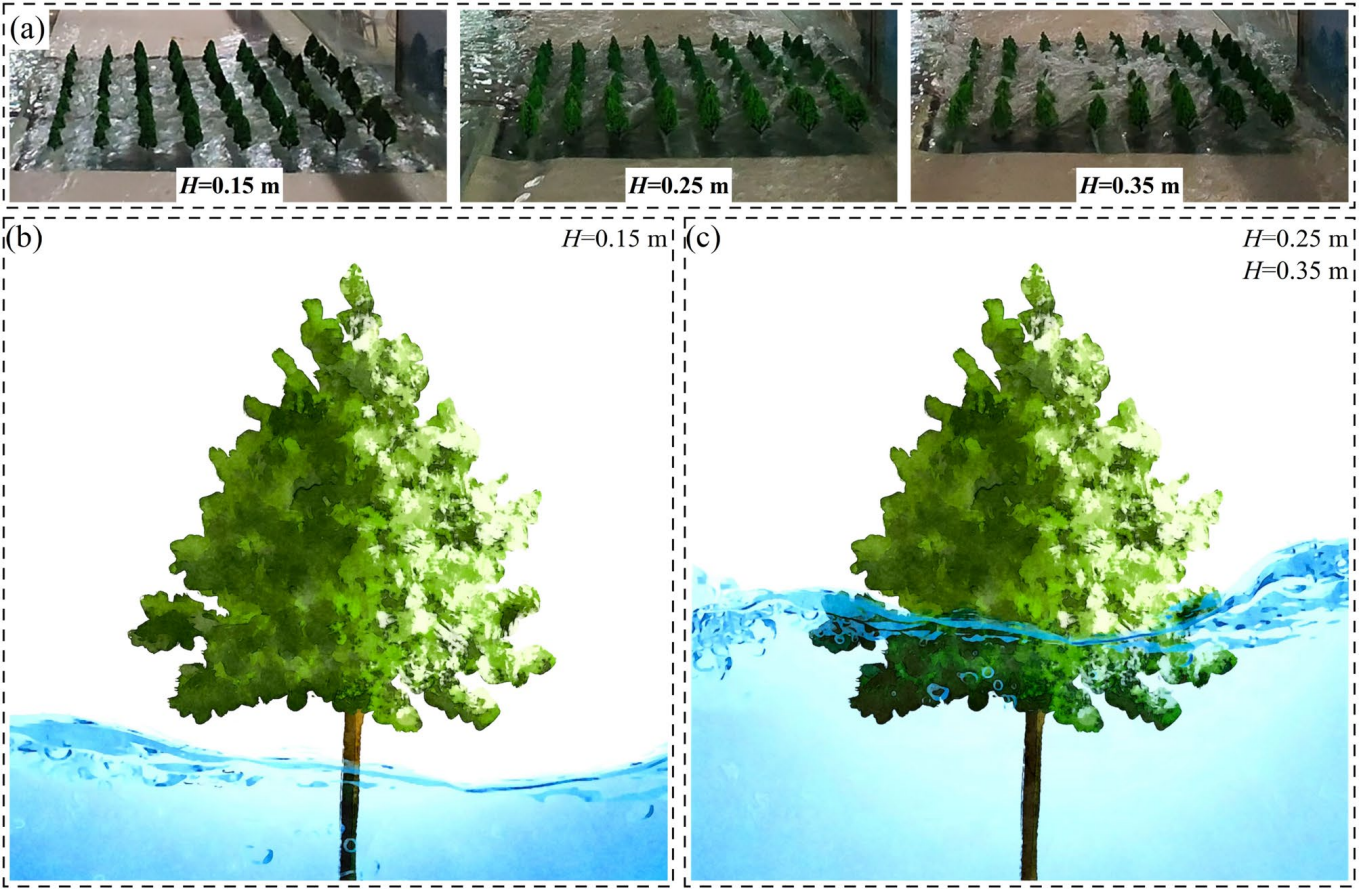
Tree-induced flow resistance and energy dissipation model.

The buffering and protective effect of tree groups on the urban area is reflected in the obstruction and energy dissipation of the floodwater by tree trunks and canopies, as shown in Fig. 8. In this experiment, there are three different levels of reservoir storage heights for the flood:

1. When the reservoir storage height is *H* = 0.15 m, the water depth around the tree groups is lower than the height of the tree trunks, and different tree arrangements have little difference in their protective effect on the urban area.
2. When the reservoir storage heights are *H* = 0.25 m and 0.35 m, the tree groups mitigate the adverse impact of the flood on the downstream urban area through the obstruction and energy dissipation provided by the tree trunks and canopies.

**Figure 9:**
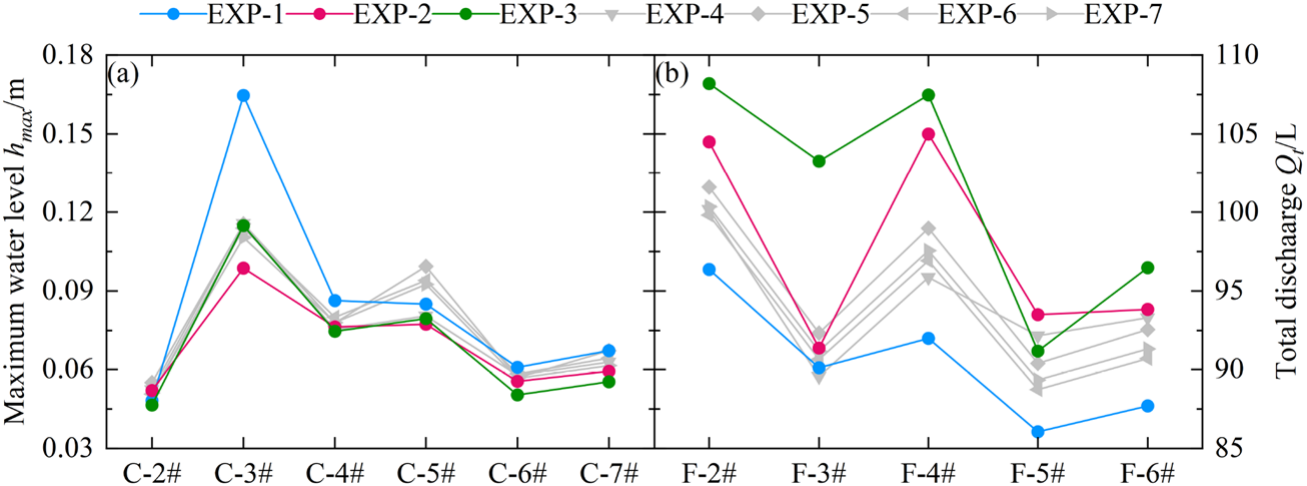
Comparison of peak water levels and drainage flow rates in different arrangements of trees.

To further investigate the buffering and protective effect of different arrangements of tree groups on floodwater, we take the example of a reservoir storage height of *H* = 0.35 m for the breach flood. Figure 9 illustrates the peak water levels measured by ultrasonic water level probes and the discharge of the pressurized stormwater drainage system during the flood propagation process in the urban street for experimental conditions EXP-1 to EXP-7, covering a time period from 0 to 300 s. Among them, the arrangement of tree groups can be determined based on the trees height: EXP-2 and EXP-3 represent tree groups arranged along the *Y*-axis, EXP-4 and EXP-5 represent tree groups arranged along the *X*-axis, and EXP-6 and EXP-7 represent tree groups arranged in both *X* and *Y* directions simultaneously.

The peak water levels at various measurement points in the urban area generally follow a trend of "increasing first and then decreasing". Among them, the water level peaks at the first column of buildings (*H*1 and *H*5) facing the incoming flood are the highest among all the experimental conditions, with measurement point C-3# reaching as high as 16.47 cm in condition EXP-1. The total discharge of rainwater at the rainwater outlets *S*1 to *S*5 shows a trend of “three peaks and two valleys”. The tributary pipes *F*-2# to *F*-11# collectively divert 904.47 L of floodwater into the mainline pipe under condition EXP-1, accounting for 19.36% of the initial reservoir volume.

Along the Y direction, the tree group arranged in an array exhibits better flow resistance and energy dissipation. This is manifested in conditions EXP-2 and EXP-3, where the peak water levels at various measurement points are smaller compared to other conditions. Additionally, the total discharge of the tributary pipes is higher in these conditions. For example, in condition EXP-2, the peak water levels at *C*-3# and *C*-4# are 9.87 cm and 7.72 cm, respectively. In condition EXP-3, the total discharge of the tributary pipes *F*-2#, *F*-3#, *F*-4#, and *F*-6# is 104.48 L, 91.37 L, 104.98 L, and 93.83 L, respectively.

During extreme flood conditions, the urban area primarily relies on the tree canopy to mitigate the adverse impacts of flood propagation. Among the four types of trees, Class *IV* trees have the largest canopy, making their placement crucial. In this study, Class *IV* trees were arranged along the *Y* direction in EXP-2 and EXP-3. These trees were strategically positioned on the upstream side during the flood propagation process, effectively reducing the kinetic energy of the floodwaters. Although Class *IV* trees were also arranged along the *Y* direction in EXP-6 and EXP-7, they were only placed in a single row, resulting in a lower protective capacity compared to EXP-2 and EXP-3.

The Yellow River, known as an overland river, has experienced over 1500 breaches in its lower reaches throughout history, with more than 20 significant channel changes. Dike-break floods can easily inundate the canopies of local tree groups in the vicinity^47^. Therefore, taller trees can be strategically planted along the river’s course and alongside streets to reduce water levels near buildings close to the breach and enhance the overall discharge capacity of the stormwater drainage system. However, the Yellow River, classified as a primary river with a high sediment load^48^, requires considerations of sediment transport in the progression of dike-break floods within the urban area. This includes assessing the sediment deposition around tree groups and the sediment-to-water ratio at stormwater inlets.

## Conclusion

This paper simulated and predicted the inundation process of dike-break flood in urban street using a generalized sink model. The key physical quantities of water level field in the study area and stormwater drainage system were measured using ultrasonic water level probes, flowmeters, and manometers. The impacts of different arrangements of tree groups on peak water levels in the urban street and discharge capacity of the pressurized stormwater drainage system were compared and analyzed. The following conclusions were drawn:

1. During the flood propagation in the urban area after the dike breach, there is a migration and variation of large-scale hydraulic jumps. The tree groups effectively prevent the occurrence of thin water layers near the buildings in the first column. Under the condition of a reservoir storage height (*H*) of 0.15 m, the maximum and minimum peak water levels at location *C*-3# for different scenarios are 5.99 cm and 4.03 cm, respectively.
2. The arrangement of tree groups enhances the flood-carrying capacity of the urban road network and further improves the discharge capacity of the pressurized stormwater drainage system. The flow conditions at the grate inlets pattern between weir flow and orifice flow during the dike-break flood propagation. The pressure fluctuations in the mainline pipe exhibit an overall maximum increase of 4.52% during the hydraulic transition process of the tributary pipes.
3. Tree groups arranged in the direction perpendicular to the flood propagation effectively reduce the peak water level in the urban area and enhance the discharge capacity of the pressurized stormwater drainage system. Under the condition of a reservoir water level of H = 0.35 m, the maximum difference in peak water levels within the urban area can reach 6.60 cm, and the total discharge volume of the pressurized stormwater drainage system increases from 3404.47 to 3513.13 L.

## Data Availability

The datasets used and analysed during the current study available from the corresponding author on reasonable request.

## References

[1] Karmakar S (2022). Urban flood risk mapping: A state-of-the-art review on quantification, current practices, and future challenges. Adv. Urban Des. Eng. Perspect. India.

[2] Yin J (2015). A review of advances in urban flood risk analysis over China. Stoch. Environ. Res. Risk Assess..

[3] Manandhar B (2023). Urban flood hazard assessment and management practices in South Asia: A review. Land.

[4] Ajjur SB (2022). Exploring urban growth–climate change–flood risk nexus in fast growing cities. Sci. Rep..

[5] Vorogushyn S (2012). Analysis of a detention basin impact on dike failure probabilities and flood risk for a channel-dike-floodplain system along the river Elbe. Ger. J. Hydrol..

[6] Kakinuma T (2014). Large-scale experiment and numerical modeling of a riverine levee breach. J. Hydraul. Eng..

[7] Rifai I (2019). Flow and detailed 3D morphodynamic data from laboratory experiments of fluvial dike breaching. Sci. Data.

[8] Zhang JH (2014). Experimental study of flood diversion in the middle and lower Han River, China. Can. J. Civ. Eng..

[9] Xia JQ (2023). Experimental and numerical model studies of dike-break induced flood processes over a typical floodplain domain. Nat. Hazards.

[10] Rong Y (2023). An improved subgrid channel model with upwind-form artificial diffusion for river hydrodynamics and floodplain inundation simulation. Geosci. Model. Dev..

[11] Testa G (2007). Flash flood flow experiment in a simplified urban district. J. Hydraul. Res..

[12] Soares-Frazão S (2008). Dam-break flow through an idealised city. J. Hydraul. Res..

[13] LaRocque LA (2013). Experiments on urban flooding caused by a levee breach. J. Hydraul. Eng..

[14] Li QJ (2023). Risk assessment of individuals exposed to urban floods. Int. J. Disaster Risk Reduct..

[15] Dong BL (2022). Risk assessment for people and vehicles in an extreme urban flood: Case study of the “7.20” flood event in Zhengzhou, China. Int. J. Disaster Risk Reduct..

[16] Dong BL (2022). Integrated modeling of 2D urban surface and 1D sewer hydrodynamic processes and flood risk assessment of people and vehicles. Sci. Total. Environ..

[17] Musolino G (2022). Enhancing pedestrian evacuation routes during flood events. Nat. Hazards.

[18] Li QJ (2022). Hazard and vulnerability in urban inundated underground space: Hydrodynamic analysis of human instability for stairway evacuation. Int. J. Disaster Risk Reduct..

[19] Guo P (2018). Selection of optimal escape routes in a flood-prone area based on 2D hydrodynamic modelling. J. Hydroinform..

[20] Xia JQ (2022). A unified formula for discharge capacity of street inlets for urban flood management. J. Hydrol..

[21] Xia JQ (2023). A unified discharge capacity formula of clogged grate inlets. Urban Water J..

[22] Guo S (2021). Experimental study of the hydraulic performance of continuous transverse grates. J. Irrig. Drain. Eng..

[23] Ekmekcioğlu Ö (2023). Exploring the practical application of genetic programming for stormwater drain inlet hydraulic efficiency estimation. Int. J. Environ. Sci. Technol..

[24] Dong BL (2021). Experimental and numerical model studies on flash flood inundation processes over a typical urban street. Adv. Water Resour..

[25] Zhang ZY (2022). Can the Sponge City Project improve the stormwater drainage system in China?—Empirical evidence from a quasi-natural experiment. Int. J. Disaster Risk Reduct..

[26] Xie K (2023). Assessment of the joint impact of rainfall characteristics on urban flooding and resilience using the Copula Method. Water Resour. Manag..

[27] Dong X (2017). Enhancing future resilience in urban drainage system: Green versus grey infrastructure. Water Res..

[28] Yazdanfar Z (2015). Urban drainage system planning and design–challenges with climate change and urbanization: A review. Water Sci. Technol..

[29] Rentachintala LRNP (2022). Urban stormwater management for sustainable and resilient measures and practices: A review. Water Sci. Technol..

[30] Singh A (2023). Drainage representation in flood models: Application and analysis of capacity assessment framework. J. Hydrol..

[31] Tavakolifar H (2021). Development of 1D–2D urban flood simulation model based on modified cellular automata approach. J. Hydrol. Eng..

[32] Luo PP (2022). Urban flood numerical simulation: Research, methods and future perspectives. Environ. Model. Softw..

[33] Hao XL (2021). Comparison of dynamic flow interaction methods between pipe system and overland in urban flood analysis. Sci. Rep..

[34] Fathy I (1974). The negative impact of blockage on storm water drainage network. Water.

[35] Tu MC (2018). Clogging impacts on distribution pipe delivery of street runoff to an infiltration bed. Water.

[36] Alves R (2022). Flood risk assessment and emergency planning—A short review. Occup. Environ. Saf. Health.

[37] Qin HP (2013). The effects of low impact development on urban flooding under different rainfall characteristics. J. Environ. Manag..

[38] Hernes RR (2020). Assessing the effects of four SUDS scenarios on combined sewer overflows in Oslo, Norway: Evaluating the low-impact development module of the Mike Urban model. Hydrol. Res..

[39] Ferguson C (2020). The impact of natural flood management on the performance of surface drainage systems: A case study in the Calder Valley. J. Hydrol..

[40] Sañudo E (2022). Comparison of three different numerical implementations to model rainfall-runoff transformation on roofs. Hydrol. Process..

[41] Dong BL (2020). Experimental investigation of flood inundation over typical urban streets. J. Hydroelectr. Eng..

[42] Lauber G (1998). Experiments to dambreak wave: Horizontal channel. J. Hydraul. Res..

[43] von Hafen H (2019). Gate-opening criteria for generating dam-break waves. J. Hydraul. Eng..

[44] Li QJ (2022). Near-field flow dynamics of grate inlets during urban floods. Phys. Fluids.

[45] Fang HY (2022). 3D CFD simulations of air-water interaction in T-junction pipes of urban stormwater drainage system. Urban Water J..

[46] Zhang, X. L. *et al*. Experimental study on the evolution of dike-break flood in urban neighborhood under pressurized stormwater pipe network[J/OL]. *Advanced Engineering Sciences*. 1–9. http://kns.cnki.net/kcms/detail/51.1773.tb.20230202.1424.003.html (in Chinese, 2023).

[47] Li T (2020). Yellow River flooding during the past two millennia from historical documents. Prog. Phys. Geogr..

[48] Wang ZC (2023). Analysis of water and sediment characteristics of the yellow river and their correlations. Pol. J. Environ. Stud..

